# Foot plantar pressure and centre of pressure trajectory differ between straight and turning steps in infants

**DOI:** 10.1038/s41598-023-34568-z

**Published:** 2023-05-16

**Authors:** Carina Price, Eleonora Montagnani, Christopher Nester, Stewart C. Morrison

**Affiliations:** 1 Human Movement and Rehabilitation Research, University of Salford, PO41 Brian Blatchford Building, Frederick Road Campus, Salford, M66PU UK; 2 School of Health Sciences, University of Brighton, 204 Aldro Building, 49 Darley Road, Eastbourne, BN20 7UR UK; 3 School of Allied Health Professions, University of Keele, Staffordshire, ST5 5BG UK; 4 School of Life Course and Population Sciences, Faculty of Life Sciences and Medicine, King’s College London, Guy’s Campus, London, SE1 1UL UK

**Keywords:** Musculoskeletal system, Paediatric research

## Abstract

Plantar pressure has been used to understand loading on infant feet as gait develops. Previous literature focused on straight walking, despite turning accounting for 25% of infant self-directed steps. We aimed to compare centre of pressure and plantar pressure in walking steps in different directions in infants. Twenty-five infants who were walking confidently participated in the study (aged 449 ± 71 days, 96 ± 25 days after first steps). Plantar pressure and video were recorded whilst five steps per infant were combined for three step types: straight, turning inwards and outwards. Centre of pressure trajectory components were compared for path length and velocity. Pedobarographic Statistical Parametric Mapping explored differences in peak plantar pressure for the three step types. Significant differences were identified primarily in the forefoot with higher peak pressures in straight steps. Centre of pressure path was longer in the medial–lateral direction during turning (outward 4.6 ± 2.3, inward 6.8 ± 6.1, straight 3.5 ± 1.2 cm, *p* < .001). Anterior–posterior velocity was higher in straight steps and medial–lateral velocity highest turning inwards. Centre of pressure and plantar pressures differ between straight and turning steps with greatest differences between straight and turning. Findings may be attributed to walking speed or a function of turning experience and should influence future protocols.

## Introduction

Gait development represents a unique phase where infants begin to move independently, explore their physical and social environments, and do so in complex ways^1^. As they do so, higher loads are applied more frequently to the foot^2^ and for longer total durations. Ambulation occurs through complex and changing patterns of movement under the management of a developing motor control system. Yet the application of forces during these tasks is critical to the shape and structure of the foot^3,4^ and forms the basis for our understanding of typical developmental pathways for attainment of ambulatory skills. Characterising the pressures on the feet at various stages of gross-motor development reflects the infant’s interaction with the support surface and has been the primary means by which gait and foot development has been studied.

Loading on the infant foot has been characterised using contact area, peak plantar pressures and force time integrals for anatomical regions of interest e.g. the forefoot^5^ and at various phases of gait development^6–9^. However, importantly, this has only ever involved infants walking under direction and in a straight line. This approach facilitates data collection and analysis, and is typical for investigation of adult walking^10^. This is at the cost of external validity of data because infants employ shorter bouts of steps in multiple directions^11,12^, with one quarter of steps involving turning once walking confidently^2^. The importance of this disparity has been discussed at length^10^ and means we are yet to fully understand what load infant feet experience during walking development.

Recent work demonstrated that, as infants become more confident, they take more steps and that proportionately more of these steps are during turning and less in a straight line^2^. This work described, but did not compare, plantar pressures under the infant foot during different types of step^2^. We therefore do not know how straight-line, inward and outward turning steps differ during infant walking in terms of plantar pressure or centre of pressure. It is therefore important that we improve the external validity of our understanding of the demands placed on foot structures as walking develops by improving our research protocols. This will also impact future academic research and clinical data collection protocols in ways that allows for greater sensitivity in detection of normal, developing and pathologic gait.

Walking turning is a complex task, involving deceleration, control of foot placement and requires substantial demand from the motor control and stability systems^13^. Difficulty during turning is well reported in individuals who may have impaired balance^14,15^ and children with cerebral palsy^16^. Previous research in adults has used the displacement and velocity of centre of pressure (CoP) trajectories as an indicator of gait and foot function, including stability^17,18^. CoP trajectory during turning to the inside and outside has been compared in adult turning tasks^19^. The authors identified a lateral and anterior shift in the CoP path when turning to the inside of the foot and a posterior shift when turning to the outside of the foot^19^. This suggests that CoP displacement is influenced by the type of walking task being undertaken, hence demands on the motor control and stability systems changes as stepping strategies change from straight to turning. Alongside CoP characteristics, turning also results in alterations to pressures experienced on the plantar surface of the feet^19,20^. These plantar pressures have been explored by researchers, in adults, reporting also contrasting outcomes. Authors agree that plantar pressures in turning steps differ to straight lines walking steps, however different experimental methods (turning angle, speed, footwear) make comparisons challenging^19,20^.

The aim of this research was to compare the CoP and plantar pressure distribution for different step directions, in confident walking infants. Therefore to explore the 25% of foot pressure data that we have ignored thus far. Describing and comparing these different steps will offer the first data set which identifies the different loading and stability demands in confident infant walkers as they move around in real-world patterns.

## Methods

Ethical approval was obtained from the School of Health Science committees at the Universities of Salford (HSCR161779) and Brighton (LHPSCREC 17–11) for a four visit longitudinal study, which was part of the Great Foundations project^21^. Parents provided informed consent for participation in the project on behalf of their infant. All methods were performed in accordance with the Declaration of Helsinki. This current work reports data from participants fourth (last) visit from our longitudinal study, at which infants were walking confidently. Confident walking was self-reported by parents who confirmed that the infant was able to walk in a stable manner, with arms by their side on multiple terrains and while holding objects and navigating more complex tasks. Researchers maintained communication with parents throughout the study and viewed videos of the infant(s) to determine readiness for participation. We sampled from the entire data set of visits available (N = 110) at random and included participants who had at least five steps of each type. Steps could be from either foot of the infant and all were included and collated as ‘steps’ with no reference to whether they were left or right. Once we reached a sample of N = 25 infants we defined our data set for this research, with this number based on a-priori sample size calculations for discrete plantar pressure analysis e.g. peak pressure in the forefoot differing between step types in our prior published work^2^.

### Testing procedure

Infants attended our child-friendly laboratory space within 21 days of achieving confident walking. The data collection areas included an embedded plantar pressure platform (size: 1.5 × 0.5 m, resolution: 4 sensors cm^−2^, frequency: 100 Hz; Novel Emed-xl, Germany) and synchronised video cameras (frequency: 50 Hz; Vicon Bonita 720c or Logitech HD Pro Webcam)^21^. Infants were given time to familiarise with the researchers and the laboratory then encouraged to walk around the area for up to 10 min while pressure and video data were collected in 60 s trials. Infants undertook self-directed playing on the mat or walking to their parent, as well as more directed tasks where they were verbally encouraged to carry a toy to the researcher.

### Data treatment

**Figure 1:**
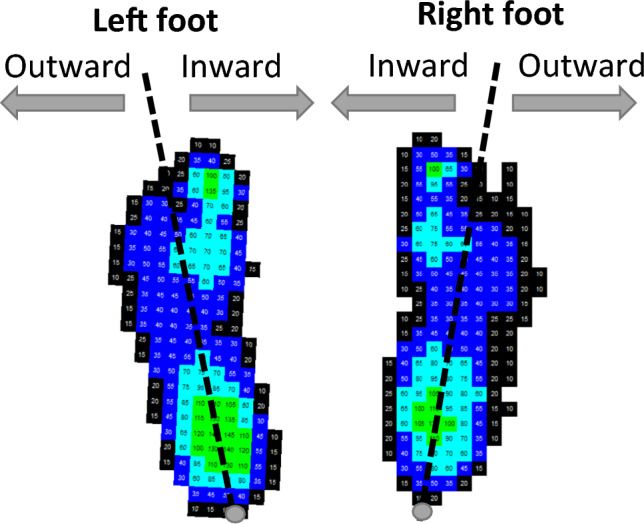
Definition of turning steps for both left and right feet as inward or outward based on the direction of the turn relative to the longitudinal axis of the foot. As defined in Price et al. (2022).

Pressure data was extracted using Emed® software (Novel gmbh, Munich, Germany) and has been previously described^2^. All steps were extracted if whole foot contact was within the platform area. Steps were defined using visual observation and coding of video data into three types: walking straight, turning inward or turning outward. This was based on the direction of the step relative to the midline along the longitudinal axis of the foot^2^. Steps were defined as:

- Walking straight: walking in a line without changing direction
- Walking curved or turning walking: walking while following a curved path or turning (changing direction). Steps were then further defined based on the direction of the turn relative to the central midline along the longitudinal axis of the foot (Fig. 1):
  o Turning inwards: towards the inside of the foot being placed/towards the opposite foot.
  o Turning outwards: towards the outside of the foot being placed/away from both feet.

Data from 10 infants was used to determine the reliability of step definition as walking straight or turning, with high reliability between two researchers (ICC_3,1_ range 0.77–0.99, median 0.98).

### Data processing

#### Peak pressure processing

Five maximum pressure pictures (MPP) of each step type were exported as ASCII files from the Novel Emascii software (Munich, Germany), and were imported into MATLAB 2019a (The Mathworks Inc, Natick, USA) as 2-dimensional (2D) numerical matrices. Non-zero entries of each matrix corresponded to pixels containing peak pressure values. Each matrix was positioned in a grid of 36 × 25 pixels. Next, right MPPs were mirrored as left using a built-in MATLAB function that returns new right-to-left MPPs matrices. MPPs were binarized, rotated to vertical with principal component analysis (PCA), transformed into point clouds and then registered within infant. Next, a mean point cloud per infant for each step type was estimated and between infant registration was undertaken, resulting in a mean MPP for each step type and for each infant. These steps have been described by Montagnani et al.^22^.

#### CoP processing

The same MPPs used for pSPM analysis were used for CoP processing. However, the individual frames composing each pressure picture were exported as ASCII format for CoP processing and were imported into Matlab as individual 2D matrices. Each individual frame was cleaned from zero entries and positioned in a grid of 36 × 25 pixels then vertically rotated using PCA^22^. Frames were combined resulting in a 3-dimensional (3D) structure for the stance phase of each step. Medio-lateral (ML) and anterior–posterior (AP) trajectories were calculated from this using footPress^23^ then interpolated to 101 points.

### Data analysis

#### Peak pressure analysis

Non-parametric SPM1D^24^ between the registered mean MPP for each step type was undertaken^25^, as required for pressure comparisons due it’s 2D nature. This implemented non-parametric one-way ANOVA and post-hoc nonparametric t-test with Bonferroni corrected p value (*p* = 0.017). A *t*-value threshold was calculated at each point of the point clouds, defining an SPM t-curve. If this threshold was crossed at any point, we were able to locate significant points^26^. This would denote peak pressure that in those points are significantly different from the paired comparisons of step type (e.g. inward turning step v outward turning step).

#### CoP analysis

Calculation of total path length and mean velocity of the ML and AP CoP trajectories utilised calculations from Quijoux et al.^27^. Data was non-parametric therefore comparison was undertaken in Statistical Software Package for the Social Sciences (SPSS statistics 26, IBM, New York, USA) using Friedman tests followed by Wilcoxon-signed rank test to isolate paired differences. P-values were based on p < 0.05 then Bonferroni corrected for multiple comparisons.

## Results

Demographic and anthropometric information of the 25 infants are presented in Table 1, six of whom were male.

**Table 1:** Demographic and anthropometric information for participants.

Characteristic	Mean	Standard deviation	Minimum	Maximum
Duration since first steps (days)	96	25	62	163
Age (days)	449	71	292	610
Mass (kg)	10.5	1.3	7.9	13.9
Height (cm)	76.0	3.6	68.8	85.0
Foot width (cm)	5.1	0.4	4.3	6.0
Foot length (cm)	11.6	0.9	9.7	13.6

### Peak pressure

**Figure 2:**
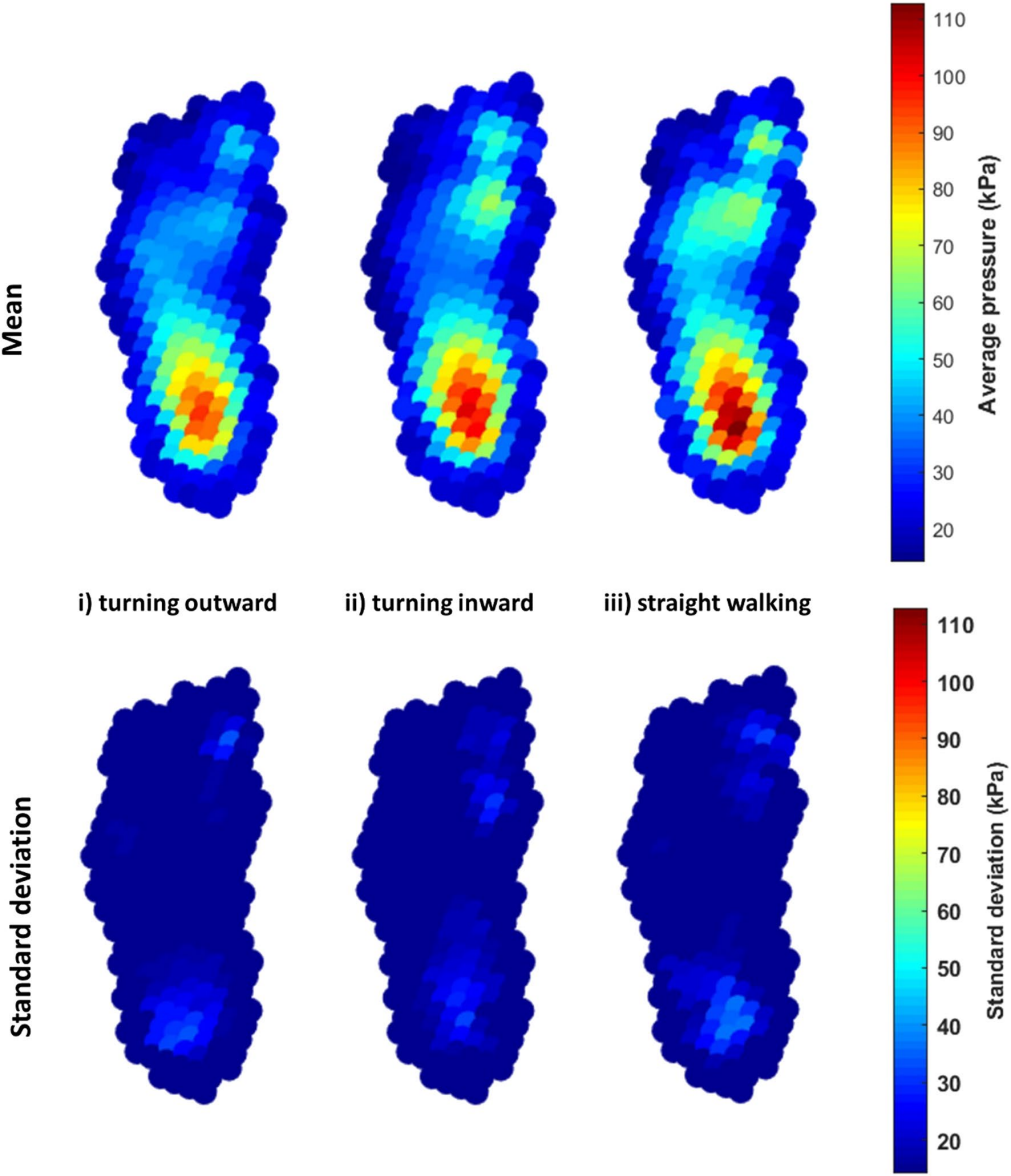
Mean and SD maximum pressure pictures for all participants for 3 step types (i) turning outward, (ii) turning inward and (iii) straight walking.

**Figure 3:**
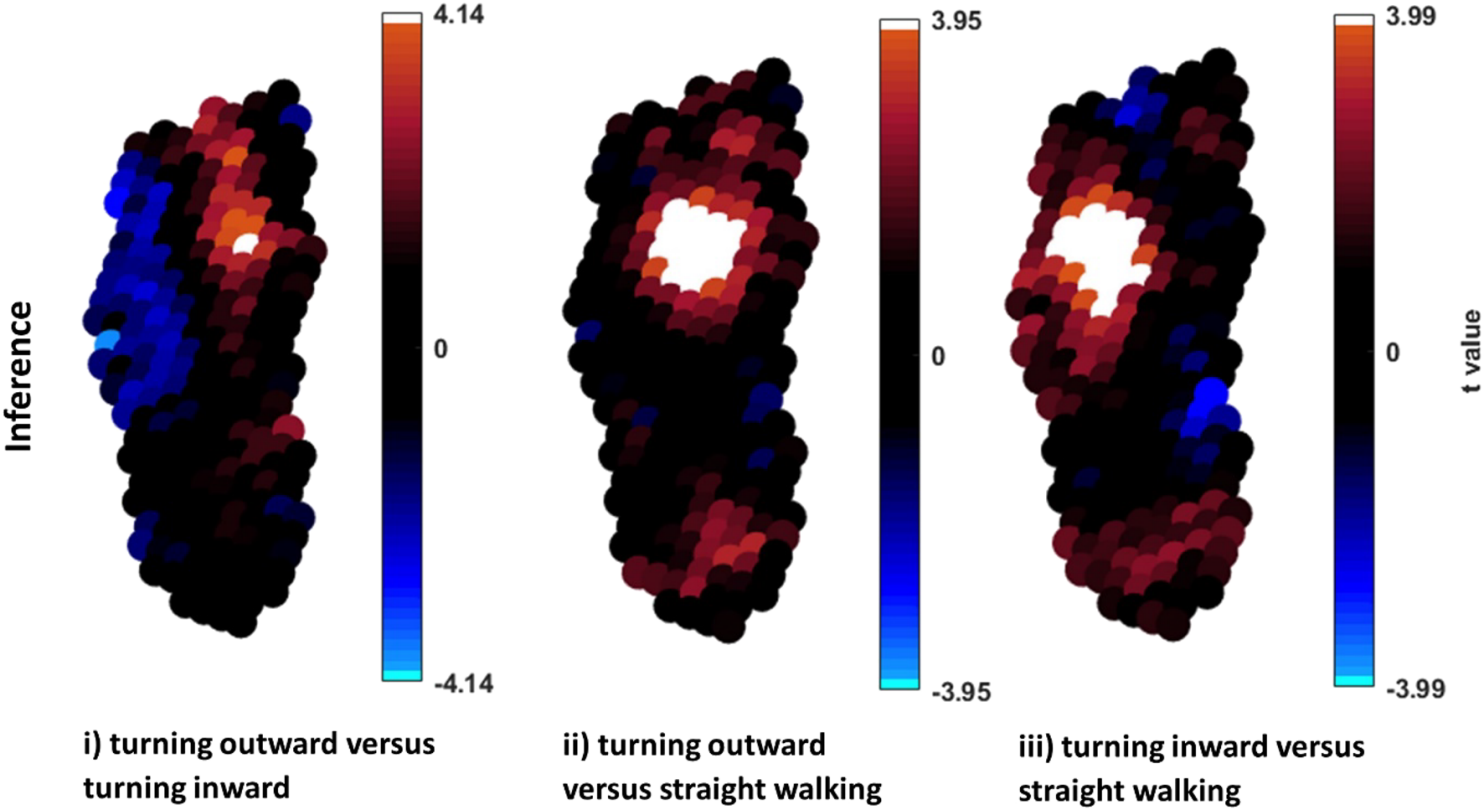
Paired samples t-test statistic SPM (t) for turning outward versus turning inward (i), turning outward versus straight walking (ii) and turning inward versus straight walking (iii). (i) The critical threshold + /- 4.14 was exceeded in a small area in the medial forefoot, where turning inward was higher than turning outward. (ii) The critical threshold± 3.95 was exceeded in a large area in the central forefoot, where straight walking was higher than turning outward. (iii) The critical threshold ± 3.99 was exceeded in a large area in the central and medial forefoot, where straight walking was higher than turning inward.

Peak pressure values differed significantly between the three step types in the forefoot region (Figs. 2 and 3). There was significantly higher pressure in a small region of the medial forefoot during inward turning compared to outward turning. (Fig. 3i). Outward turning involved significantly higher pressure in the central forefoot, and inward turning higher pressure in the lateral forefoot, compared to straight walking (Fig. 3ii,iii).

### CoP

The ML path length was significantly longer in the turning inward steps than turning outward and straight walking (Table 2). The AP velocity was higher in straight walking than during turning, contrasting ML velocity which was highest in the inward turning steps (Table 2).

**Table 2:** Centre of pressure path lengths and velocities for straight and turning steps (median (IQR)) with significant outcomes.

		Turning outward (TO)	Turning inward (TI)	Straight walking (SW)	*Significant differences*
Path length (cm)	AP	14.5 (6.9)	11.4 (10.2)	13.4 (4.8)	*p* = *.326*
	ML	4.6 (2.3)	6.8 (6.1)	3.5 (1.2)	*TI* > *TO, p* < *.001* *TI* > *SW, p* < *.001*
Mean velocity (cm s^−1^)	AP	17.7 (9.3)	17.7 (11.2)	22.8 (10.8)	*SW* > *TO, p* = *.012* *SW* > *TI, p* = *.017*
	ML	5.8 (2.3)	9.3 (8.5)	5.9 (2.5)	*TI* > *TO, p* < *.001* *TI* > *SW, p* < *.001*

Where significant differences are presented including post-hoc assessments for Turning outward (TO), turning inward (TI) and straight walking (SW).

## Discussion

This study reveals that peak plantar pressures and CoP trajectories differ between straight, inward, and outward turning steps during walking in infants. For plantar pressure, significant differences between the three step types were identified in the forefoot region, with the greatest differences between both turning steps and straight-line walking, and least differences between inward and outward turning steps. For the CoP data, differences were most notable between the straight and turning steps. Even in the very early stages of walking, the data suggests that there are changes in how the foot is interacting with the ground according to the task at hand. This means that if we want to understand the development of gait in infancy, and particularly the demands being placed on the foot, we cannot just observe their straight-line walking.

It might be assumed that the greatest differences would be between outward and inward turning steps, since these are functionally opposite tasks for the foot, however only a small area of peak pressure under the medial/central forefoot of the infants differed (higher pressure for inward turning). Similarly, in the CoP data, the ML path length and velocity were the only differences between turning steps (higher for inward). AP characteristics of the CoP were consistent between turning steps, but differed when compared to straight steps. These outcomes may be a function of the infants learning to turn and therefore not having turning strategies which are specific to the direction of the turn at this stage^13^. Furthermore, previous research considering turning has either controlled^28^ or recorded^13^ the turning strategy used. Observing our self-directed participants (an approach we use to preserve ecological validity), the turning strategies were mixed, with turns sometimes encompassing walking on an arc for numerous consecutive steps, and other times spinning on the spot on one foot. These strategies were also reflected in different body positions, arm positions and velocities within-strategy. This is consistent with gait in infants being highly variable inter and intra-individual^29^ and even walking in a straight line being a result of multiple strategies which are experience dependent^30^. Consistent with this idea, the standard deviation of the CoP data was far higher in turning steps than steps that were in a straight line for path length. This inter-individual variability is expected and reflects both different strategies for turning and variability in performing turns, likely a function of the inexperience of this cohort performing a relatively new skill^31^. Similarly, this mirrors high variability and different strategies to achieve turning in other populations who report difficulty turning^15^.

In the present study, plantar pressures were lower during turning steps than during straight line walking. Lower pressures were distributed in the central metatarsals in outward turning steps and lower pressure in the central and lateral metatarsal regions in inward turning steps both compared to straight line walking. These lower pressures might be due to lower walking velocity when turning as they occur in foot regions where pressure is influenced by walking speed in children^32^. A previous study has reported a significant increase in central forefoot pressures in infants when they were running compared to when they were walking^33^, reinforcing that the foot areas we identified changes are those where peak pressures are influenced by walking speed. We did not record walking speed, but the AP velocity of the CoP has previously been used as a marker for walking speed^17^ and was significantly lower in both turning steps compared to straight line walking. Furthermore, in adults, previous literature reports that turning steps are slower than straight line walking steps when undertaken at self-selected speeds^19^. In infancy, this difference in speed may also be enhanced by turning being a new skill and further challenging motor control, potentially requiring the skill to be performed more slowly for success. Therefore, we would anticipate both turning steps to be slower in infants and this change in movement speed to reduce the pressure experienced under the central forefoot.

This association between the forefoot (central metatarsals in particular) pressures and walking speed and/or turning is likely associated with the role of the forefoot during propulsion. Reduced peak power generation at the ankle has been reported in adults taking turning steps, attributed to a reduced requirement for forward acceleration^34^, which we would anticipate to be reflected with a reduction in forefoot pressure at push-off. However, this pattern was not mirrored in children (aged 11 ± 3 years), where ankle powers were consistent between straight line and turning steps^28^. The authors speculated that ankle powers assisted turning and propelling the body in the new walking direction^28^, contrasting findings in adults. The relevance of the forefoot push-off to generating forces is yet to be defined in this infant population due to less potential power generation at the foot/ankle^35^. The forefoot has an important role in infancy when walking in a straight line in early stance, particularly in new walkers, as it offers a large contact area for initial foot contact with the ground^5,36^. It is likely that this role is maintained in turning steps. We acknowledge the limitations with the data reported in this study and appreciate that kinematic data would have offered insight. The comparison of data using pSPM to compare peak pressure values from the whole of stance also limits our interpretation of data as it loses its temporal aspect, and thus, we do not know at which frame in stance these peak pressures are occurring. Further research would benefit from a temporal analysis throughout stance to separate the roles of the forefoot in early and late stance in infancy.

The high ecological validity of our protocol ensured infants self-selected their movements and therefore their straight line and turning steps were performed without restrictions. This led to some constraints in experimental design and the feasibility of outcome variables meaning that we were limited to peak pressure and centre of pressure comparisons for interpretation. Furthermore, this free-movement approach meant that the infant turning steps were uncontrolled and undefined in terms of their method of turning^13^, severity of angle or phase of turn in which they were extracted from the turning process e.g. preparation to turn, recovery from turn^28,37^. As aforementioned, this has led to high variability in our data and potentially masked differences between the inward and outward turns. The lack of difference between these step types may also be due to our selection of variables and our limited sample size. However, the method we chose did not impose constrained turns on these individuals, which may not reflect their variable level of accomplishment of the turn in their real-world environment. In addition to allowing the infants to self-direct when and how they turned, we also let them self-select their walking speed and did not have a method to capture this. Therefore, we can only make predictions that some of the differences we have identified between step types are due to differences in walking speed when turning.

## Conclusions

Through adopting a data collection strategy that enabled infants the freedom to explore and move within a safe environment, our work advances understanding of plantar pressures in infancy. The pressure on infant feet differs depending on the direction of the step they are taking. Pressure is significantly higher in the forefoot when walking in a straight line than both turning inwards and outwards. Inward turning steps had significantly higher pressure in a small region of the medial forefoot than outward turning steps. This data is the first to consider real-world complexities of early walking and we hope that other researchers and clinicians adopt a more externally valid approach to gait assessment in infancy.

## Data Availability

The datasets used and/or analysed during the current study available from the corresponding author on reasonable request.
